# Evaluation of the correlation between dosimetric, geometric, and technical parameters of radiosurgery planning for multiple brain metastases

**DOI:** 10.1002/acm2.13326

**Published:** 2021-07-01

**Authors:** André Vinícius de Camargo, Minsong Cao, Diego da Cunha Silveira Alves da Silva, Raphael Leonardo Cunha de Araújo

**Affiliations:** ^1^ Department of Radiation Oncology Barretos Cancer Hospital Barretos Brazil; ^2^ Department of Radiation Oncology University of California Los Angeles CA USA; ^3^ Barretos Cancer Hospital Barretos Brazil; ^4^ Universidade Federal de São Paulo São Paulo Brazil; ^5^ Hospital Israelita Albert Einstein São Paulo Brazil

**Keywords:** dosimetrics parameters, geometrics parameters, multiple brain metastases, radiosurgery, technical parameters

## Abstract

**Purpose:**

To evaluate the correlation between dosimetric, geometric, and technical parameters for radiosurgery planning of multiple brain metastasis treatments treated with a linear accelerator with volumetric modulated arc therapy (VMAT) technique.

**Materials and methods:**

Data were collected retrospectively from 55 patients who underwent radiosurgery in a single institution from August 2017 to February 2020. Patients presented 4–21 brain metastases were treated with a single fraction with doses between 18 and 20 Gy. Dosimetric variables were collected including V5Gy, V8Gy, V10Gy, V12Gy, V14Gy, conformity index (CI), heterogeneity index (HI), maximum dose (Dmax), and the CI_R50. Geometric variables including the number of lesions, target volumes, the smallest target volume, the largest target volume, and the distance between the isocenter and the most distant lesion (DIL) and technical variables such as the numbers of total arcs, noncoplanar arcs, and isocenters were collected for analysis.

**Results:**

The number of lesions had a moderate positive correlation with V5Gy, V8Gy, V10Gy, V12Gy, V14Gy, HI, Dmax, and with the number of total arcs. The target volumes had a positive medium–high correlation with V5Gy, V8Gy, V10Gy, V12Gy, V14Gy, and moderate positive correlation with HI, Dmax, number of arcs and noncoplanar arcs. The CI and CI_R50 had a negative correlation with all volumes related to the target: the target volumes, the smallest, and the largest lesion. A positive correlation was observed between the distance of the isocenter and the most DIL with V5Gy, V8Gy, V10Gy, V12Gy, V14Gy, HI, Dmax, and the number of isocenters.

**Conclusion:**

It was found that the number of lesions and the target volumes are good predictors of dosimetric indexes of plan evaluation and that the distance between the isocenter and the most DIL harms them.

## 1 INTRODUCTION

Stereotactic radiosurgery (SRS) is a noninvasive procedure that uses ionizing radiation to treat intracranial lesions with an ultrahigh dose of radiation delivered in a single fraction of treatment. As a result, the planning and delivery of the dose must be extremely accurate.

The two main commercially available dose delivery methods of SRS are based on either dedicated radisurgery systems such as Gamma Knife (GK) and Cyber Knife or conventional medical linear accelerators (LINAC) with cone or micro‐multileaf colimator (MLC), and image‐guided radiatiotherapy (IGRT).^1^, ^2^ One study showed that there are statistically significant differences between treatments with GK and LINAC, and specifically with LINAC, there is also a difference in the plan of dosimetric quality achieved between the treatment planning systems (TPS) (BrainLab Elements, Eclipse® and HyperArc) and between different planners using the same TPS.^3^

Recently, LINAC‐based SRS has been gaining more and more interests in the radiosurgery treatment for multiple lesions, because in addition to achieving great results of planning and dose delivery, it is a widely available equipment capable of treating small lesions intracranial and extracranial. Another advantage of LINAC based on SRS is a shorter treatment time.^3^

Many authors look for intrinsic factors of the case such as number of lesions, target volumes, and shapes and characteristics of planning^4^, ^5^ that can influence plan evaluation indexes such as the dose that the healthy brain receives, conformity index (CI), and others. There is also a search for an equation that relates these factors to dosimetric parameters, as it already exists for a single lesion.^6^

Recently, studies have focused on the dosimetric and clinical impact of the use of multiple isocenters when treating multiple lesions^7^, ^8^, ^9^, ^10^ with volumetric modulated arc therapy (VMAT), mainly caused of rotational errors of positioning and image fusion, and how to solve this problem ^11^ but still with no consensus on the subject.

Thus, this study aims to assess the correlation between dosimetric, geometric, and technical parameters for planning multiple brain metastasis treatments treated with a linear accelerator using the VMAT technique.

## 2 MATERIALS AND METHODS

Retrospective plan dosimetric data were collected from 55 patients who underwent radiosurgery at Barretos Cancer Hospital from August 2017 to February 2020. Patients who presented within 4–21 brain metastases delineated with the aid of a magnetic resonance images (MRIs) were treated in a single fraction with a dose of 18 or 20 Gy. Frameless immobilization system was used for simulation and treatment. Simulation computed tomography (CT) with 1.25 mm of slice thickness was used for all plannings.

All patients were treated on a Varian TrueBeam® ™ STX Varian Medical Systems linear accelerator with high definition MLC of 120 leafs and planned with Eclipse® TPS (Varian Medical System Inc, version 13.6). The calculation algorithm used was the Anisotropic Analytical Algorithm (AAA) with a 1.25‐mm calculation grid and heterogeneity correction. The plans were optimized by different physicists according to their experience and the needs of each case, but all sought to achieve objectives established by the department of coverage of targets and dose constraints in the organs at risk according to the radiosurgery protocol for brain metastases, as described in the Supporting Information. The beam setup and the number of isocenters were defined by the physicist who planned each case. VMAT (RapidArc®, Varian Medical system, Inc.) treatment technique was used for all cases with a planning target volume (PTV) margin of 1 mm from gross tumor volume (GTV) contour.

Three groups of plan variables, dosimetric, geometric, and technical data, were restropectively collected. Dosimetric variables included were V5Gy, V8Gy, V10Gy, V12Gy, V14Gy, CI, heterogeneity index (HI), Dmax, and the 50% isodose CI (CI_R50). The VxGy represents the volume of the “x” Gy dose that the normal brain minus PTV received. The CI was calculated by the ratio between the volume of the prescription isodose and the volume of the PTVs: $V_{presc_{\mathrm{isodose}}/V_{\mathrm{PTVs}}}$. The HI was calculated as${\;D}_{2\%}‐D_{98\%}/D_{50\%}$.^12^ Dmax is the maximum point dose of the plan, and the CI_R50 is the ratio between the volume of the 50% isodose line and the volume of the PTV.

In the group of geometric variables were collected data concerning the number of lesions, total target volumes, the smallest and the largest target volumes, and the distance between the isocenter and the most distant lesion (DIL). The distance between the isocenter and the most DIL was determined using the coordinates of the lesion center and its respective isocenter. The calculation was according to Equation (1). In cases where there were more than 1 isocenter, the distance was measured between the isocenter and the most DIL that its arcs treated.

(1) $$d=\sqrt{{\left(X_{\mathrm{Lesion}}‐X_{\mathrm{Iso}}\right)}^{2}+{\left(Y_{\mathrm{Lesion}}‐Y_{\mathrm{Iso}}\right)}^{2}+{\left(Z_{\mathrm{Lesion}}‐Z_{\mathrm{Iso}}\right)}^{2}}.$$

Finally, technical variables included were total number of arcs, coplannar or noncoplanar arcs, number of noncoplanar arcs if used, and number of isocenters.

For statistical analysis, Spearman's rank correlation coefficient (CC) was calculated to determine the degree and direction (directly or inversely proportional) of the correlation between the numerical variables of the three groups. This coefficient varies between −1 and +1 in which these two extremes indicate a strong correlation. The negative and positive signs show that the variables are inversely and directly proportional, respectively. The Mann–Whitney test was used to determine whether there was a statistically significant difference between the groups using or not noncoplanar arcs in relation to the dosimetric and geometric variables. The Kruskal–Wallis test was used to analyze variance in categorical variables in relation to dosimetric and geometric variables. It was considered statistically significant when *p* < 0.05. All data were collected and managed through the REDCap platform.^13^

Data are available at the request from authors.

## 3 RESULTS

### 3.1 Descriptive statistics

Descriptive analyses of the geometric, dosimetric, and technical variables are summarized in Table 1. Briefly, the mean number of lesions was 6.58, ranging from 4 to 21 lesions. The average volume of the targets was 6.41 cc, but the median was 3.84 cc. The mean DIL was 6.01 cm, and the maximum was 9.3 cm. The average of the V12 Gy was 12.91 cc, and the maximum was 35.1 cc.

**TABLE 1:** Descriptive statistics of geometric, dosimetric, and technical variables

Variables		Mean	Standard deviation	Median	Minimun	Maximun
Geometric	Number of lesions	6.58	3.90	5.00	4.00	21.00
	Target volumes (cc)	6.41	6.11	3.84	0.72	22.94
	Smallest target volume (cc)	0.21	0.17	0.15	0.02	0.68
	Largest target volume (cc)	3.10	3.76	1.50	0.17	16.69
	DIL (cm)	6.01	1.50	6.20	1.70	9.30
Dosimetric	V5 Gy (cc)	126.16	118.00	88.80	29.70	772.80
	V8 Gy (cc)	38.23	27.68	29.10	11.70	177.10
	V10 Gy (cc)	21.07	12.08	16.70	7.30	63.80
	V12 Gy (cc)	12.91	6.93	10.40	4.60	35.10
	V14 Gy (cc)	7.85	4.05	6.30	2.80	19.40
	CI	1.20	0.20	1.17	0.91	2.10
	HI	13.53	3.29	13.90	7.00	20.00
	Dmáx (%)	118.76	4.65	118.70	109.40	130.10
	R50	8.95	3.12	7.95	4.20	17.65
Technical	Number of arcs	4	2	4	2	8
	Number of noncoplanar arcs	2	1	2	1	4

Abbreviations

CI, conformity index; DIL, distance between the isocenter and the most distant lesion;HI, heterogeneity index.

Technical data on the use of coplanar arcs and the number of isocenters are listed in Table 2. It was found that non‐coplannar arcs were used in a large fraction of the treatment plans (67.3%), and 4 isocenters were used in one case.

**TABLE 2:** Descriptive statistics of technical variables

Variables	Options	Count	Percentage
Noncoplanar arcs?	Yes	37	67.3%
	No	18	32.7%
Number of isocenters	1	42	76.4%
	2	10	18.2%
	3	2	3.6%
	4	1	1.8%

**FIGURE 1:**
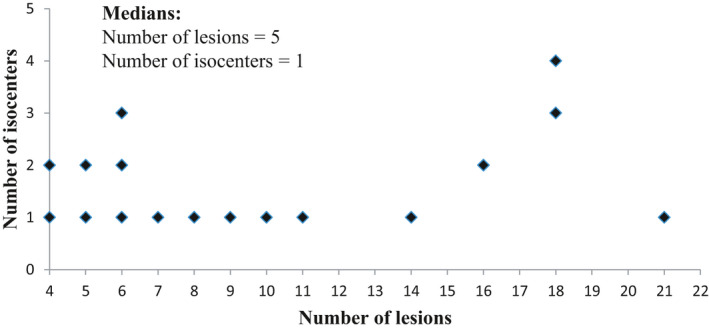
Relationship between the number of lesions and the number of isocenters

The relationship between the number of lesions and the number of isocenters can be seen in Figure 1.

### 3.2 Dosimetric versus geometric variables

The CCs between the dosimetric and geometric variables that had statistical significance (*p* < 0.05) are provided in Table 3. It is possible to notice that there are numerous correlations (strong and weak) between the variables of the two groups.

**TABLE 3:** Correlation coefficient between geometric and dosimetric variables that had statistical significance (*p* < 0.05)

Dosimetric variables	Geometric variables				
	Number of lesions	Target volumes	Smallest target volume	Largest target volume	DIL
V5 Gy	0.586	0.827	–	0.708	0.424
V8 Gy	0.533	0.862	–	0.762	0.393
V10 Gy	0.494	0.866	–	0.783	0.383
V12 Gy	0.469	0.857	–	0.777	0.375
V14 Gy	0.420	0.819	–	0.738	0.341
CI	–	−0.355	−0.371	−0.373	–
HI	0.347	0.325	–	0.289	0.334
Dmáx	0.280	0.411	–	0.393	0.313
CI_R50	–	−0.519	−0.549	−0.554	–

Abbreviations

CI, conformity index; DIL, distance between the isocenter and the most distant lesion;HI, heterogeneity index.

The number of lesions had a moderate positive correlation with the V5Gy, V8Gy, V10Gy, V12Gy, V14Gy, HI, and Dmax, and it means as bigger the number of lesions, the greater are values of these variables. The target volume had a positive medium–high correlation with the V5Gy, V8Gy, V10Gy, V12Gy, and V14Gy and a moderate positive correlation with the HI and Dmax.

In addition, correlation analyses were performed between the number of lesions and V5Gy, V8Gy, V10Gy, V12Gy, and V14Gy of patients grouped into five groups categorized by the target volumes (0 < V ≤ 2, 2 < V ≤ 3, 3 < V ≤ 6, 6 < V ≤ 10, and 10 < V ≤ 25 cc). Significant strong positive correlations were found between the number of lesions and V5Gy (CC =0.645), V8Gy (CC =0.731), and V10Gy (CC =0.683) within the group with a target volumes between 0 < V ≤ 2 cc and also strong positive correlations between the number of lesions and V5Gy (CC =0.786), V8Gy (CC =0.744), V10Gy (CC =0.777), and V12Gy (CC =0.758) within the group with a target volumes between 2 < V ≤ 3 cc. The groups were categorized so that each group had approximately 11 patients.

**FIGURE 2:**
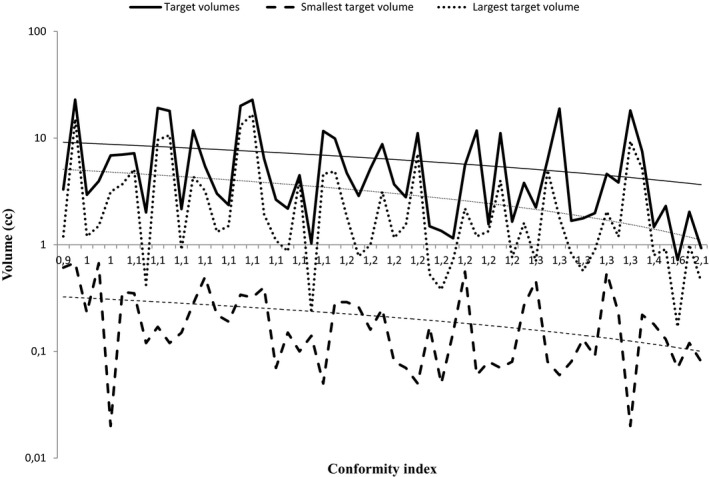
Relationship of volumes with the conformity index (CI). The *y*‐axis is on a logarithmic scale

The CI and CI_R50 had a weak negative correlation with all volumes related to the targets: the target volumes, the smallest, and the largest target. It means that CI and CI_R50 are inversely proportional to these variables. The behavior of these volumes in relation to the CI can be seen in the graph in Figure 2. The *y*‐axis is on a logarithmic scale. Note that the trend lines are decreasing, as previously suggested by Stanley et al.^14^

**FIGURE 3:**
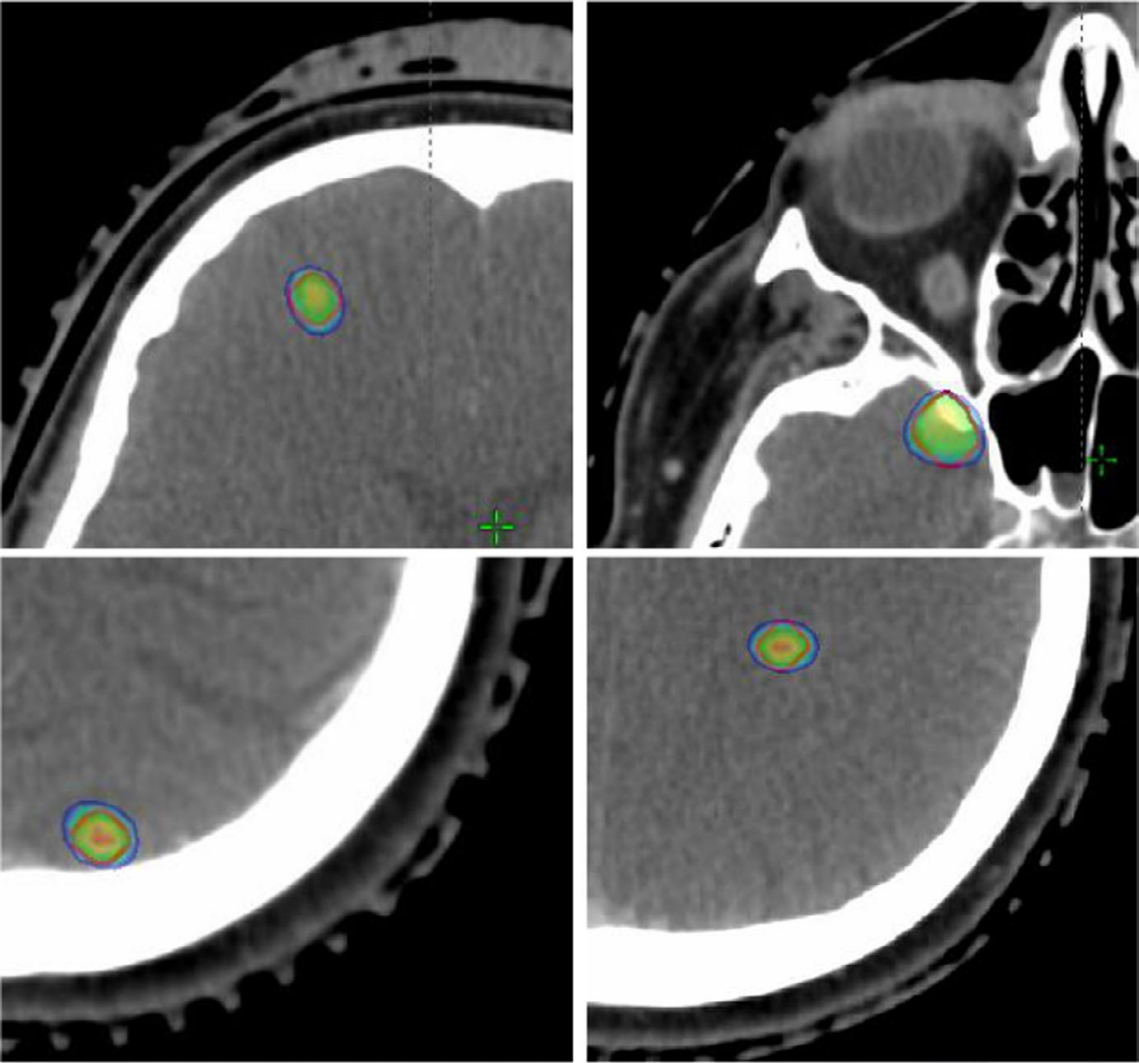
Dose distribution in the four lesions in the case with CI = 2.1

As depicted in Figure 2 and demonstrated in Table 1, there was a case with CI = 2.1. This extreme case corroborates the weak negative correlation obtained that states that the higher the CI, the lower the volume of the targets (0.93 cc). The dose distribution for the four lesions in this particular case is demonstrated in Figure 3. It is possible to observe that the prescription dose exceeds the limits of PTV in all lesions.

The relationship of the V12 Gy and the CI_R50 with the target volumes is listed in Table 4.

**TABLE 4:** Behavior of the CI_R50 and the V12 Gy in relation to target volumes

Target volumes (cc)	CI_R50			V12 Gy (cc)		
	Minimun	Median	Maximum	Minimum	Median	Maximum
0 < V ≤ 2	7.9	11.0	17.7	4.6	6.7	8.4
2 < V ≤ 3	5.6	9.4	13.6	6.9	8.9	13.2
3 < V ≤ 6	5.7	7.6	10.2	7.7	12.3	10.2
6 < V ≤ 10	4.6	6.9	8.0	8.6	16.7	22.4
10 < V ≤ 25	4.2	7.3	15.4	8.5	22.6	35.1

**FIGURE 4:**
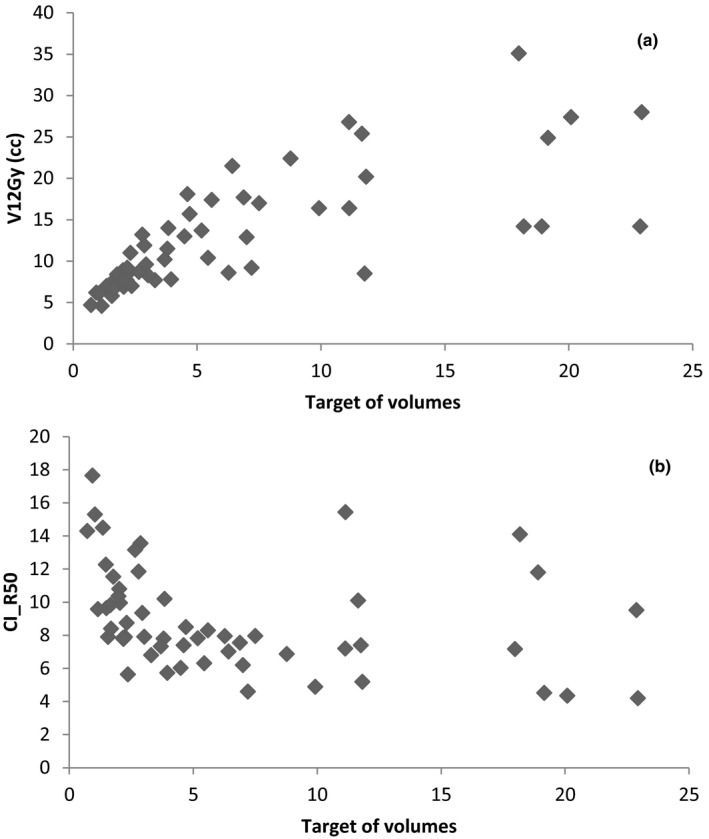
Graph a shows the relationship between the target volumes and the V12 Gy. Graph b shows the relationship between the target volumes and the CI_R50

The data in Table 4 are also illustrated in Figure 4. As greater were the targets volume, greater were also the V12Gy, and the reverse occurs with the CI_R50. As depicted in Figure 4b, an increase in CI_R50 after 10 cc was found. Due to the small number of cases with volumes greater than 15 cc, it is not possible to conclude what the real trend of the CI_R50 would be for these volumes. Moreover, the data in Table 4 show that the V12Gy was not always below 10 cc, as several authors suggest as an important constraint for normal brain tissue.^15^, ^16^

Finally, a weak positive correlation was found between the distance of the isocenter and the most DIL with V5Gy, V8Gy, V10Gy, V12Gy, V14Gy, HI, and Dmax. This means that the greater the distance, the greater the volumes that the healthy brain receives at certain doses and the greater the heterogeneity of the plan.

### 3.3 Geometric versus technical variables

The CCs between the geometric and technical variables that had statistical significance are shown in Table 5. The differences between groups that used noncoplanar arc or not and between groups based on number of isocenters in relation to the geometric variables were also demonstrated in Table 5.

**TABLE 5:** Correlation coefficient between geometric and technical variables that had statistical significance (*p* < 0.05)

Geometric variables	Technical variables			
	Number of arcs	Noncoplanar arcs?	Number of noncoplanar arcs	Number of isocenters
Number of lesions	0.443	–	–	–
Target volumes	0.285	^a^	0.412	–
The smallest target volume	–	–	–	–
The largest target volume	–	–	–	–
DIL	–	–	–	^b^

Abbreviations

DIL, distance between the isocenter and the most distant lesion.

^a^ There was a difference between the two groups (yes × no) in relation to the geometric variable.

^b^ There was a difference between the groups (1, 2, 3, and 4 isocenters) in relation to the geometric variable.

The number of lesions had a moderate positive correlation with the number of arcs, which means that more arcs were used in cases where there were more lesions. The volume of the targets also showed a moderate positive correlation with the number of arcs and the number of noncoplanar arcs; that is, the greater the volume of the targets, the more arcs were used and the greater the number of noncoplanar arcs.

A statistically significant difference between the groups that used or not noncoplanar arc in relation to the target volumes was found. It suggests that the decision to use or not to use noncoplanar arc are related to the target volumes. The median number of lesions and the target volumes of the cases in which it was or was not chosen to use a noncoplanar arc are shown in Table 6, and difference between the two groups was noted. Through it is possible to observe that it was chosen to use noncoplanar arc mainly in cases where there were more lesions and in which the target volumes were greater, and in the latter, it is seen that the volume, when using noncoplanar arc, it is almost twice the volume compared to cases in which a noncoplanar arc was not used.

**TABLE 6:** Variables, number of lesions, and target volumes for cases in which noncoplanar arc was used and for those in which it was not used.

	Noncoplanar arc?	
	Yes	No
	Median	Median
Number of lesions	6.0	4.5
Target volumes (cc)	4.7	2.5

A point to be observed in Table 5 is that the distance between the isocenter and the most DIL was not correlated with number of arcs, number of noncoplanar arcs, or the fact of using or not noncoplanar arc but only with the number of isocenters. Focusing only on 1 and 2 isocenters, through Table 2, it is possible to conclude that this correlation is inversely proportional, which means that the greater the number of isocenters, the smaller the distance. The number of cases with 3 and 4 isocenters is very low in relation to the other two, so it may not be representative, even so, we see that there was a case that even using 4 isocenters, the longest distance between one of them and the most DIL was 9.3 cm. This need was due to the high number of injuries, 18, the large target volumes, 18.2 cc, and the distribution of them in the brain that were scattered in all directions.

### 3.4 Dosimetric versus technical variables

The CCs between the number of arcs and the dosimetric variables obtained statistical significance and are listed in Table 7.

**TABLE 7:** Correlation coefficient between the number of arcs and the dosimetric variables that had statistical significance (*p* < 0.05)

Dosimetric variable	Number of arcs
V5 Gy	0.300
V8 Gy	0.285
V10 Gy	–
V12 Gy	–
V14 Gy	–
CI	–
HI	–
Dmáx	–
CI_R50	–

Abbreviations

CI, conformity index; HI, heterogeneity index.

The number of arcs had a weak positive correlation with the V5Gy and the V8Gy. This is justified due to the fact that the number of arcs also increases with the number of lesions and with the volume of lesions, as described in Section 3.3. Moreover, it seems more consistent observing the number of lesions, which is the parameter determinant for the use of more arcs, because the greater the number of lesions, the more dispersed they are throughout the brain, thus requiring a larger number of arcs, as detailed in Table 8.

**TABLE 8:** Number of injuries and target volumes with respect to the number of arcs used

Number of arcs	Frequency	Number of lesions	Target volumes
		Median	Median (cc)
2	6	4.0	2.4
3	10	4.0	2.2
4	19	6.0	5.2
5	9	6.0	1.8
6	6	7.5	9.1
7	1	6.0	2.3
8	4	12.0	9.3

A statistically significant difference was found in whether or not to have a noncoplanar arc for the variables V10Gy, V12Gy, V14Gy, and CI_R50. This difference can be found in Table 9.

**TABLE 9:** Dosimetric variables that had a statistically significant difference when using or not using noncoplanar arc (*p* < 0.05)

Dosimetric variables	Yes	No
	Median	Median
V5 Gy (cc)	–	–
V8 Gy (cc)	–	–
V10 Gy (cc)	21.00	12.20
V12 Gy (cc)	13.00	7.80
V14 Gy (cc)	7.90	4.90
CI	–	–
HI	–	–
Dmáx (%)	–	–
CI_R50	7.82	9.89

Abbreviations

Abbreviations: CI, conformity index; HI, heterogeneity index.

It should be noted that the normal brain volumes that receive 10, 12, and 14 Gy were greater when using noncoplanar arc. However, as shown in Table 6, the median number of lesions and the target volumes were higher in cases using noncoplanar arcs; this demonstrates that it was chosen to use noncoplanar arc mainly in the most complex cases, and so the values of the V10Gy, V12Gy, and V14Gy were higher. In Section 3.3, it was shown that there is a positive correlation between the target volumes and the number of noncoplanar arcs, corroborating this trend. Therefore, it should not be concluded that the V10Gy, V12Gy, and V14Gy would be larger when using noncoplanar arcs, because in our department, noncoplanar arcs are used when coplanar arcs do not achieve good results, as well as recent studies suggest that noncoplanar arcs can achieve better planning results.^17^, ^18^, ^19^, ^20^, ^21^

Observing the CI_R50, it was found that when using noncoplanar arc, its value is lower, exactly what is sought in radiosurgery planning.

## 4 DISCUSSION

Regarding the correlation between the number of lesions and volumes (V5Gy, V8Gy,…), considering the greater the number of lesions, more areas of the brain are involved in planning making it more difficult to spare the normal brain. Thus, the worsening heterogeneity of the plan is justified.

The reduced number of isocenters used in planning with VMAT, which at the time of delivery of the dose is an advantage, as it reduces the treatment time, in planning can become a limiting factor, because with multiple injuries, the isocenter can stay away from one or more lesions, thus increasing the field size of the arcs, this, in addition to being able to exceed the mechanical range of the leaves, can force the system to use the thicker collimator leaves, losing spatial resolution, thus making optimization of the plan difficult.

**FIGURE 5:**
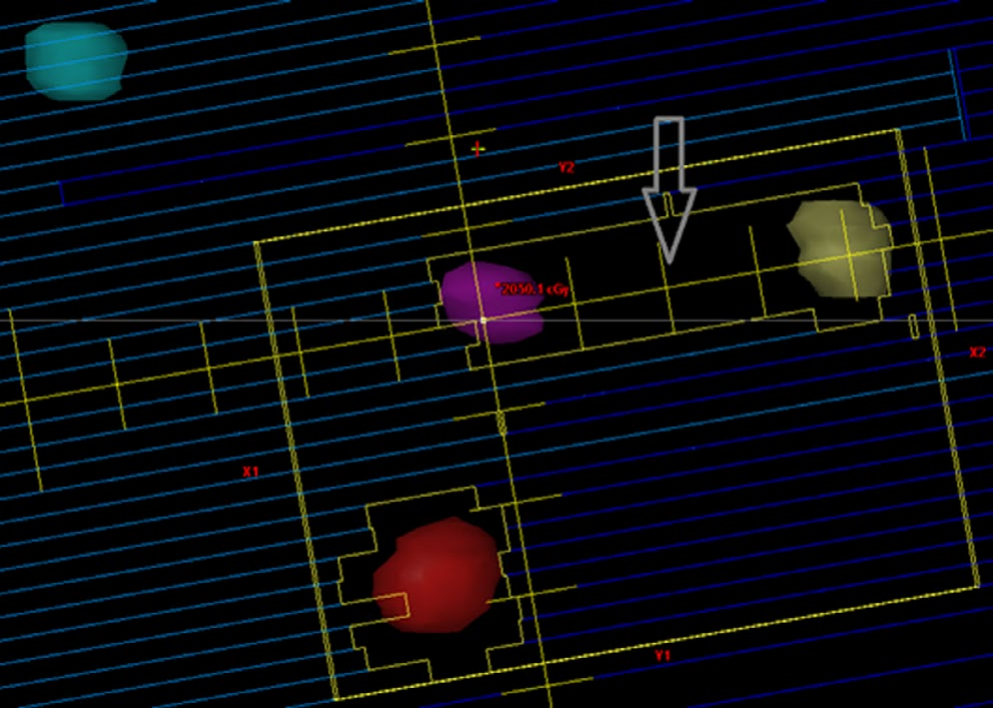
Beam's eye view of the multileaf colimator (MLC) of an arc

Another difficulty point is the presence of multiple lesions, because two or more targets could be overlapped in the direction of the leaves’ movement, creating areas where the leaves would need to be closed but will remain open so as not to collimate one or more injuries (Figure 5). Consequently, the increasing of the number of lesions growths the volume of doses and the heterogeneity of the plan.

The number of lesions also had a moderate positive correlation with the number of arcs. The more lesions there are, more scattered they are, and more complex the case is, thus, greater degrees of freedom are required. However, it does necessarily mean that it is necessary to place a very large number of arcs in order for the plan to reach all objectives; as shown in Table 1, 8, eight was the largest number of arcs used.

Regarding the correlation between the target volumes and the dosimetric volumes mentioned above, it is known that the target volumes can be increased in two ways: by increasing the number of lesions, as previously discussed above, or, if the lesions are larger, as the volume of the prescription isodose increasing proportionally to the volume of the lesions, the lower doses isodoses (5, 8, 10, 12, and 14 Gy) also increase affecting a greater volume of the healthy brain. Concerning the correlation between the target volumes and the HI and the Dmax, when the volume is increased due to the increase in injuries, the arguments have already been presented. The correlation between these variables was also found by Narayanasamy et al.^4^

The volumes of the smallest and largest lesions had the same behavior as the target volumes in relation to the dosimetric variables with which they had a statistically significant correlation; that is, when the volume of the lesions had a positive correlation with some dosimetric variable, the volume of the smallest and the largest lesions also had it, and this also occurred when there was a negative correlation. This is consistent because both the volume of the smallest injury and the volume of the largest injury are contained within the target volumes.

Regarding the correlation between the number of lesions and the dose volumes that the normal brain receives, it is noted that when performing the test including all patients, a moderate correlation was found, and when the patients were grouped, some groups had a strong correlation, and others, with a target volumes greater than 3 cc, did not show a statistically significant correlation. This may have occurred due to the small number of patients in each group and the large difference in target volumes within groups with volumes greater than 3 cc, especially in the 10‐ to 25‐cc group.

The distance between the isocenter and the most DIL behaved exactly like the number of lesions, correlating with the same variables. As previously discussed, when the number of lesions increases, the distance between the isocenter and the lesions increases (because the aim is always to use the smallest possible number of isocenters), and this ends up hampering the optimization, thus harming the healthy brain volumes receiving the specified doses and heterogeneity of planning.

In addition, as it is already known^7^, ^8^, ^9^, ^10^ that small angulation errors affect the coverage of the PTV and the dose in nearby organs, and the impact of this is greater with the increase in distance; however, the work also showed that the increase in distance infers the difficulty of planning, also impacting the V5Gy, V8Gy, V10Gy, V12Gy, V14Gy, and the heterogeneity of planning. The data also showed that the distance decreases with the number of isocenters. These results suggest that the maximum distance between the isocenter and all lesions should be limited. For this, from a certain distance, a new isocenter should be used. This can improve planning results and lessen the impact of setup errors and of image fusion on the linear accelerator.

This study has some limitations, such as the plans were carried out by different planners, difference in the prescribed dose and in some patients one or more lesions (with greater volumes) were treated in 3 fractions, but even in these cases, they always had 4 or more lesions that were treated with a single dose. In the case of prescription, a statistical test was applied to check if there was a difference between the dose of 18 and 20 Gy in relation to the geometric, dosimetric, and technical variables, and no statistically significant difference was found. Morover, it is a retrospective study, and selection bias cannot be ruled out.

The CI, calculated by the department based on the definition of the Radiation Therapy Oncology Group (RTOG), has some limitations, and one of them is the dependence on the choice of the prescription isodose.^22^ Although the cases of this work were planned by different physicists, they all followed the same coverage objectives defined by the department's protocol (Supporting Information): D95% = 100% for PTVs and D100% = 100% for GTVs. The CI evaluation criteria are also those defined by the RTOG. Thus, it is expected that there will be no major discrepancies between the CI values obtained by the different planners. Even so, a CI = 2.1 was still obtained. By RTOG, if this index is between 2 and 2.5, the protocol violation is considered to be minor.

Anyway, even with these results, a more in‐depth study that relates the dosimetric indexes to toxicity should and will be carried out so that, perhaps, new limits may be determined that can be reached in cases of multiple lesions.

## 5 CONCLUSION

Through the results, it can be concluded that both the number of lesions and the target volumes are good predictors of the heterogeneity of the planning and of the volumes of the doses of 5, 8, 10, 12, and 14 Gy that the healthy brain will receive.

## CONFLICT OF INTEREST

The authors declare no conflicts of interest.

## Supporting information

Data S1. Radiosurgery protocol for brain metastases.
