# Supplementary material for: Evaluation of the correlation between dosimetric, geometric, and technical parameters of radiosurgery planning for multiple brain metastases
Source: J Appl Clin Med Phys. 2021 Jul 1;22(8):83–92. doi: 10.1002/acm2.13326 (PMC8364278; doi:10.1002/acm2.13326)
Supplement: Supplementary file 1 — Data S1. Radiosurgery protocol for brain metastases. [file ACM2-22-83-s001.pdf]

DEPARTMENT OF RADIOTHERAPY

Radiosurgery planning for brain metastases

Name: ____

Medical record: ________________________ **Technique (3D/IMRT/VMAT):** ________________________

Dose Prescription

	**PTV _____**	**PTV _____**	**PTV _____**
Dose	___ x ____ cGy = ____ Gy	___ x ____ cGy = ____ Gy	___ x ____ cGy = ____ Gy
Normalization			
Number of arc/day			
GTV	$D_{100\%} \geq 100\%$:_____	$D_{100\%} \geq 100\%$:_____	$D_{100\%} \geq 100\%$:_____
PTV	$D_{95\%} \geq 100\%$: _____<br>$D_{0.03cc} \leq 120_{(125)}\%$: _____<br>$1 < CI < 2.0 = V_{DOSE}/V_{PTV}$:_____<br>$R50 < 5.0 = V_{ISO\ 50\%}/V_{PTV}$:_____	$D_{95\%} \geq 100\%$: _____<br>$D_{0.03cc} \leq 120_{(125)}\%$: _____<br>$1 < CI < 2.0 = V_{DOSE}/V_{PTV}$:_____<br>$R50 < 5.0 = V_{ISO\ 50\%}/V_{PTV}$:_____	$D_{95\%} \geq 100\%$: _____<br>$D_{0.03cc} \leq 120_{(125)}\%$: _____<br>$1 < CI < 2.0 = V_{DOSE}/V_{PTV}$:_____<br>$R50 < 5.0 = V_{ISO\ 50\%}/V_{PTV}$:_____

	**STRUCTURE**	**CRITERIA**	**OBSERVATION**	**PRIORITY**
☐	Brain - PTVs	$V_{12Gy} \leq 10cc$: _____		
☐	Cochleas	R: $D_{0.03cc} \leq 9Gy$: _____ L: $D_{0.03cc} \leq 9Gy$: _____		
☐	Lens	R: $D_{0.03cc} \leq 2Gy$: _____ L: $D_{0.03cc} \leq 2Gy$: _____		
☐	Hippocampus	$V4.5Gy \leq 40\%$:____ $D_{0.03cc} \leq 6{,}6Gy$:____		
☐	Spinal cord	$D_{0.35cc} \leq 10Gy$:_____ $D_{1.2cc} \leq 7Gy$:_____ $D_{0.03cc} \leq 14Gy$: _____		
☐	Optic nerves	R: $D_{0.2cc} \leq 8Gy$:_____ $D_{0.03cc} \leq 10Gy$: _____<br>L $D_{0.2cc} \leq 8Gy$:_____ $D_{0.03cc} \leq 10Gy$: _____		
☐	Chiasm	$D_{0.2cc} \leq 8Gy$:_____ $D_{0.03cc} \leq 10Gy$: _____		
☐	Retinas	R: $D_{0.03cc} \leq 20Gy$: _____ L: $D_{0.03cc} \leq 20Gy$: _____		
☐	Brainstem	$D_{0.5cc} \leq 10Gy$:_____ $D_{0.03cc} \leq 15Gy$: _____		

OBS: Up to 3 lesions, complete the limits separately for each PTV. For ≥ 4 lesions, fill data for the sum of PTVs, but all lesions must respect D95% = 100% for PTV and D100% = 100% for GTV.

Physician: ________________________________ **Medical physicist:** ________________________________
